# Cardiac arrest teams perspectives on communication and ethical conflicts related to awareness during CPR, a focus group study protocol

**DOI:** 10.1186/s13049-018-0550-x

**Published:** 2018-09-27

**Authors:** Rune Sarauw Lundsgaard, Kristine Sarauw Lundsgaard

**Affiliations:** 1 Department of Anesthesiology Nykøbing Falster Hospital, Nykøbing Falster, Denmark; 2 Department of Occupational Medicine, Copenhagen University Hospital Holbæk, Holbæk, Denmark

**Keywords:** Cardiac arrest, Cardiopulmonary resuscitation, Consciousness, Awareness, Cardiac arrest teams, End-of-life care, Patient-centered approach

## Abstract

**Background:**

Awareness during Cardio Pulmonary Resuscitation (CPR) also called CPR induced consciousness (CPRIC) is a rare, but increasingly reported condition with significant clinical implications. Health professionals lack guidelines about patients with CPRIC, and to this date, no studies have addressed the complexity of communication and ethical aspects when continuing CPR while the patient is conscious.

**Methods:**

We aim to explore Cardiac arrest team members perspectives regarding communication and ethical conflicts related to awareness during CPR. We have designed a qualitative, descriptive study using focus groups to discuss and reflect on patients with awareness during CPR. Focus groups consist of cardiac arrest team members (senior and training medical doctors, nurses and hospital porters). We will be presenting already published case reports about patients with CPRIC to focus groups to facilitate discussion and debate regarding the team members perceptions. Data analysis is inductive and based on systematic text condensation.

**Discussion:**

Previous studies have suggested that external stressors affect the performance of a Cardiac arrest team. As a result of our analysis, we will aim to describe communicative and ethical challenges and concerns regarding awareness during CPR. Recent studies in the area point to a desire for guidelines and we hope to contribute with knowledge, that can inform the further process when developing guidelines and training team members to handle these stressful and important cases.

**Trial registration:**

The study involves no healthcare intervention on human participants.

## Background

Cases on patients who show signs of awareness during Cardio Pulmonary Resuscitation (CPR), also called CPR induced consciousness (CPRIC) have been reported scarcely in the medical literature [1]. However, the phenomenon has gained more attention in the past two years than ever before [2–4], and a recent cross-sectional study shows that experienced health practitioners anecdotal reports of awareness during CPR/CPRIC is far more common than reflected in the medical literature [5].

Awareness during CPR has several clinical implications, yet no guidelines address these patients and protocols, e.g., on pain management/sedation is still at an early stage [6]. Health practitioners facing these clinical scenarios experience very different management strategies and diverging clinician opinions and express the desire of a uniform management/guideline regarding both the recognition of awareness/CPRIC and resuscitative efforts, which are challenged due to patient awareness [5–7].

Apart from the clinical challenges, awareness during CPR also give rise to communicative and ethical dilemmas. No studies have yet specifically addressed these issues, but studies about “family present during CPR” as well as studies about end-of-life discussions reveals how dealing with patients emotions regarding death is challenging and can provoke anxiety in the health practitioner [8, 9]. These studies point to increased focus on in-situ debriefing of everyone involved [8, 10].

Previous studies have suggested that the technical performance by a Cardiac arrest team is affected by the presence of external stressors regardless of the experience level of the team leader [11, 12]. This stress the need for increased knowledge on how Cardiac arrest team members perceive challenges and stressors when patients show signs of awareness during CPR.

## Methods/design

### Aim and design

The study is designed as a qualitative, descriptive study, using focus groups to explore cardiac arrest team members perspectives on communication and ethical conflicts related to awareness during CPR.

### Sampling/participants

The research has been exempted from ethical approval by the Regional Ethics Committee Zealand, Denmark. Focus groups are constructed of cardiac arrest team members with varying experience and clinical background from a rural hospital in Eastern Denmark.

Participants are senior and training doctors, nurses and hospital porters. The primary researcher (RL) will inform all participants verbally and in writing and obtain written consent.

### Data collection

Focus group sessions is set to last of 30–60 min. The principal investigator RL will act as a group facilitator and use case reports from medical literature [4, 7, 13] to initiate debate about communicative and ethical aspects of awareness during CPR.

Data collection will continue until no new data is obtained on the area of interest. Informant check will be done in the form of a summative list of themes during the focus group process.

### Data processing and analysis

All focus group sessions are recorded using a digital recorder, transcribed verbatim, and anonymized and data from the discussion are used together with the summative list of themes from each session. Data analysis is carried out alongside data collection until saturation is achieved. The team responsible for data analysis and interpretation is mixed and included medical doctors with different level of experience regarding CPR as well as education scientists. The principal author (RL), who leads the data collection and interpretation, is an MD and with experience in anesthesiology, emergency medicine, and medical education.

To explore how cardiac arrest teams perceive communication and ethical conflicts when patients show awareness during CPR we will use an inductive analytic approach [14].

## Discussion

The data collection is potentially challenged by clinicians in different working hours and many challenging tasks and demands. We have tried to accommodate this by planning the study in a rural hospital, where the staff is mostly living nearby, potentially increasing the possibility of recruitment. Also, we hope that the recent attention from international media [15, 16] has evoked public interest in the subject, hopefully increasing our ability to recruit participants to our focus groups. We are aware that patients are important stakeholders in the development of guidelines and protocols, however we were not able to define one specific group of patients to include in the study, as cardiac arrest is a condition with a low rate of successful survival.

## Abbreviations

CPR: Cardio Pulmonary Resuscitation
CPRIC: Cardio Pulmonary Resuscitation Induced Consciousness
